# Dynamic causal modelling in probabilistic programming languages

**DOI:** 10.1098/rsif.2024.0880

**Published:** 2025-06-04

**Authors:** Nina Baldy, Marmaduke Woodman, Viktor K. Jirsa, Meysam Hashemi

**Affiliations:** ^1^ Aix-Marseille Universite, UMR INSERM 1106 Institut des Neurosciences des Systèmes, Marseille, France

**Keywords:** ordinary differential equations, Bayesian inference, multi-modality, dynamic causal modelling, adaptive Hamiltonian Monte Carlo, variational inference

## Abstract

Understanding the intricate dynamics of brain activities necessitates models that incorporate causality and nonlinearity. Dynamic causal modelling (DCM) presents a statistical framework that embraces causal relationships among brain regions and their responses to experimental manipulations, such as stimulation. In this study, we perform Bayesian inference on a neurobiologically plausible generative model that simulates event-related potentials observed in magneto/encephalography data. This translates into probabilistic inference of latent and observed states of a system driven by input stimuli, described by a set of nonlinear ordinary differential equations (ODEs) and potentially correlated parameters. We provide a guideline for reliable inference in the presence of multimodality, which arises from parameter degeneracy, ultimately enhancing the predictive accuracy of neural dynamics. Solutions include optimizing the hyperparameters, leveraging initialization with prior information and employing weighted stacking based on predictive accuracy. Moreover, we implement the inference and conduct comprehensive model comparison in several probabilistic programming languages to streamline the process and benchmark their efficiency. Our investigation shows that model inversion in DCM extends beyond variational approximation frameworks, demonstrating the effectiveness of gradient-based Markov chain Monte Carlo methods. We illustrate the accuracy and efficiency of posterior estimation using a self-tuning variant of Hamiltonian Monte Carlo and the automatic Laplace approximation, effectively addressing parameter degeneracy challenges. This technical endeavour holds the potential to advance the inversion of state-space ODE models, and contribute to neuroscience research and applications in neuroimaging through automatic DCM.

## 1. Introduction

Dynamic causal modelling (DCM; [1–3]) has become a cornerstone methodology in neuroimaging for understanding and interpreting complex brain dynamics. Central to DCM is Bayesian model inversion on a set of ordinary differential equations (ODEs), which aims to infer the posterior distribution of lumped parameters given the prior and observed data [4–6]. This process is challenged by the complexity and high-dimensionality of the models, often rendering evaluation of the posterior density computationally intractable [7]. To address this issue, one common approach is to use a fixed-form probability distribution to approximate the posterior density, which relaxes the challenging computation of high-dimensional integrals into an optimization problem. Traditionally, variational inference (VI; [8,9]) has been the preferred approach for this task, due to its computational efficiency in handling large-scale problems and the analytical solution it offers [10–13]. However, parametrized approaches such as VI are not without drawbacks, such as challenges in accurately approximating multi-modal posteriors and providing reliable uncertainty estimates.

In light of these limitations, efforts have been made to propose alternatives to variational inference for Bayesian model inversion in DCM [14–17]. A benchmark study was conducted using a comprehensive panel of non-parametric sampling-based methods on a neural mass model of event-related potentials (ERPs) measured in magneto/encephalography (MEG/EEG) recordings [16,17]. These studies involved custom code for implementing the tested algorithms, which were released as Markov chain Monte Carlo (MCMC) inference in the DCM reference toolbox Statistical Parametric Mapping [18,19]. Initial studies focused on gradient-free MCMC methods, including random walk Metropolis, slice-sampling, adaptive MCMC and population-based MCMC with tempering [16]. Gradient-free methods, although fast, struggled to thoroughly explore the posterior space and failed to produce a full, exploitable posterior distribution necessary for extracting key quantities, such as quantiles. Subsequent work focused on gradient-based MCMC methods, particularly emphasizing Langevin Monte Carlo [20,21] and Hamiltonian Monte Carlo (HMC; [22]) algorithms [17]. By leveraging the first-order gradients of the joint log-likelihood function, these classes of MCMC methods aim to alleviate the slow mixing problem and statistical inefficiency that plague gradient-free samplers.

Despite tremendous effort and extensive testing, the results of previous studies [16,17] were not encouraging in terms of convergence and the number of independent samples produced per unit computational time. While fitting the data was relatively achievable within an acceptable error margin, obtaining accurate inference on the neurobiological parameters of the model proved to be more difficult. Gradient-based methods suffered from prohibitively high computational costs given the relatively small effective sample size they produced. Moreover, dynamics-based methods, such as HMC, face challenges in setting algorithmic parameters such as the step size and the number of steps for their numerical integration. The No-U-Turn Sampler (NUTS; [23]) addresses these challenges by using a recursive algorithm to dynamically determine algorithmic parameters based on the geometry of the target distribution. Nevertheless, when dealing with nonlinear differential equations involving correlated parameters and partial observations, challenges related to achieving convergence within reasonable computational costs emerge.

This work aims to address the limitations encountered in previous approaches by exploring alternative programming languages and platforms for DCM implementation. It emphasizes the importance of cross-platform compatibility and accessibility in advancing neuroimaging research. Our research tackles the practical challenges of Bayesian inference in partially observable nonlinear systems of differential equations, which are fundamental tools in most scientific domains. With advancements in computation, particularly in automatic differentiation and adaptive algorithms bolstered by the progress of machine learning techniques, the high computational costs of gradient-based methods are being significantly mitigated [24]. This development could potentially facilitate the routine application of sophisticated sampling schemes in DCM, and, broadly speaking, state-space ODEs models.

One promising solution lies in probabilistic programming languages (PPLs), which are a class of programming languages designed to facilitate the specification, manipulation and inference of complex probabilistic models [25]. These languages integrate standard programming constructs with probabilistic modelling, allowing for the seamless combination of deterministic and stochastic components. PPLs simplify the process of specifying complex probabilistic models. Instead of manually deriving mathematical formulations for inference, users can write models using high-level programming syntax, which makes the modelling process more intuitive and accessible. Additionally, PPLs provide built-in inference algorithms, saving time and reducing the potential for errors compared with manual implementation. Many PPLs are optimized for performance, leveraging advanced computational algorithms, techniques and hardware acceleration to efficiently handle large datasets and complex models, ensuring scalability [26]. PPLs such as Stan [27], PyMC [28] and NumPyro [29], provide powerful tools for probabilistic modelling, offering advantages in flexibility, automated inference, integration with computation, scalability and applicability across diverse scientific domains.

We underscore the efficacy of PPLs, which, through implementation in various libraries with interfaces for common data science languages (such as Python and C++), achieve rapid fitting, high accuracy and enhanced efficiency. Our results show significant improvements in the effective sample size obtained per unit of computational time, particularly by leveraging the JAX library [30], which offers composable function transformations and automatic differentiation for high-performance machine learning. Moreover, we conduct a comparative analysis between the NUTS and the variational methods. Through this comparison, we delineate the strengths and weaknesses of each method, ultimately showcasing their convergence to the same posterior distribution when successfully applied. We conduct comprehensive model comparison using both variational inference (assessed through free-energy; [12,31]) and MCMC frameworks (evaluated with fully Bayesian information criteria; [5,32]). Our results demonstrate close alignment between methods in the MCMC framework and the free-energy obtained from variational inference, ensuring consistency in model comparison.

Importantly, another challenge for accurate DCM is the non-identifiability and its geometrical counterpart known as degeneracy, i.e. the existence of multiple equivalent solutions in the parameter space. Degeneracy is ubiquitous across biological systems [33,34], and crucial for the brain’s resilience and adaptability [35,36]. While degeneracy provides an adaptive mechanism for maintaining brain function (degeneration and dedifferentiation; [37]), allowing compensatory strategies to deal with impairments and diseases, it also exacerbates the difficulties associated with non-identifiability in parameter estimation. This interplay contributes to multi-modality in the posterior distributions, where multiple sets of parameters can equally explain the observed data. This complicates the inference process and leads to wasted computational resources. We propose remedies for practical multi-modality, which manifests as convergence to different modes in repeated inference runs, overestimation or underestimation when using variational inference methods, or poor mixing in MCMC sampling. Our solutions include (i) optimal setting of algorithmic parameters, (ii) initialization at the tail of prior distribution, and (iii) weighted stacking of chains to average Bayesian predictive distributions [38], all of which significantly improve the inference.

In brief, our investigation underscores the balance between computational expense and inferential accuracy, highlighting the critical demand for flexible, efficient and accurate Bayesian inference methods in neuroimaging. The tools are now accessible on the cloud platform EBRAINS (https://ebrains.eu), enabling users to explore more realistic brain dynamics underlying neurological conditions within a Bayesian causal framework.

## 2. Material and methods

### 2.1. Dynamic causal modelling

DCM [1–3] is a well-established statistical framework that allows for estimation of causal interactions between brain regions, based on neuroimaging data, such as functional magnetic resonance imaging (fMRI), MEG or EEG. It evaluates causal responses to diverse experimental manipulations, particularly on evoked responses to stimuli. Central to DCM is the concept of effective connectivity, which denotes the influence that one brain region or neuronal system exerts over another. It quantifies the directed interactions between distinct units, thereby delineating the causal relationships within the brain’s functional network. DCM formalizes the nonlinear dynamics of brain activity by representing them as a biologically informed nonlinear dynamical system [39]. Model inversion in DCM involves not only estimating the biophysical and effective connectivity but also enables source reconstruction through the inference of hidden states. In models of event-related potentials (ERPs), these hidden states correspond to the activity in non-observed sources [40].

### 2.2. Neural mass model

Neural mass models (NNMs; [41,42]) efficiently describe the collective behaviour and interactions of large populations of neurons under the mean-field assumptions. They are widely used to understand brain dynamics at a macroscopic scale, during resting state [43,44], task-related activities [45,46], as well as in altered states such as anaesthesia [47,48], healthy [49] and diseased conditions [50–52].

**Figure 1.**
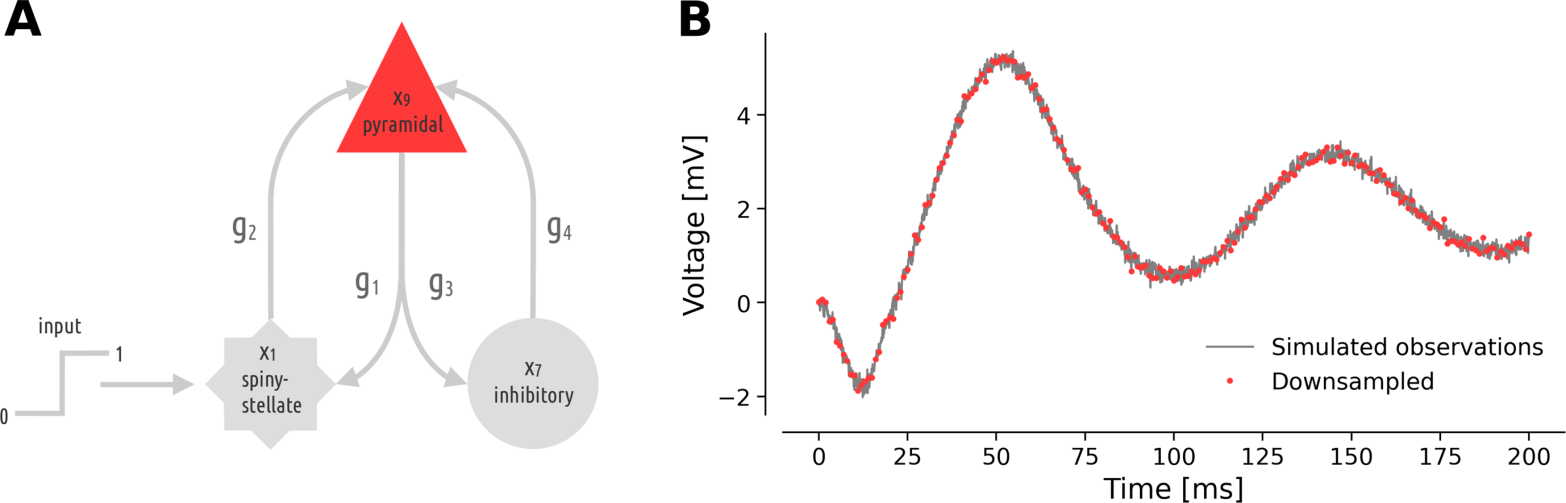
A DCM of ERPs [39]. (A) Schematic representation of the causal relations in the forward model consists of three neural populations, namely, pyramidal neurons, inhibitory interneurons and spiny-stellate cells, driven by exogenous Heaviside input, according to equation (2.1). (B) The pyramidal cell voltage with additive Gaussian noise (in grey) is the only observable in the model and was downsampled by a factor of 10 (in red).

In this study, we focused on a neurobiologically plausible generative NMM of ERPs measured with MEG/EEG recordings [39]. The model comprises three interconnected neural populations (figure 1A): spiny-stellate cells ($x_{1}$), which receive the input current, inhibitory interneuron ($x_{7}$) and the only observed population: excitatory pyramidal neurons ($x_{9}$). The temporal evolution of model variables is described by a nine-dimensional system of ODEs (given by equation (2.1)), serving as a first-order approximation to delay differential equations by $x(t-\delta )=x(t)-\delta \dot{x}(t)$. This evolution is governed by a set of 10 parameters that are described in table 1. The perturbation input to spiny-stellate cells is modelled as a Heaviside step function, where the parameter u represents its intensity [16,17],


(2.1) $$\begin{array}{lcr} & \begin{array}{rl} {\dot{x}}_{1}(t) & =x_{4}(t)\\ {\dot{x}}_{2}(t) & =x_{5}(t)\\ {\dot{x}}_{3}(t) & =x_{6}(t)\\ {\dot{x}}_{4}(t) & =\frac{h_{e}}{\tau_{e}}\left(g_{1}\left(\frac{1}{\exp^{-0.56x_{9}(t-\delta )}\;+1}-0.5\right)+u\right)-\frac{x_{1}(t)}{\tau_{e}^{2}}-\frac{2x_{4}(t)}{\tau_{e}}\\ {\dot{x}}_{5}(t) & =\frac{g_{2}h_{e}}{\tau_{e}}\left(\frac{1}{\exp^{-0.56x_{1}(t-\delta )}\;+1}-0.5\right)-\frac{x_{2}(t)}{\tau_{e}^{2}}-\frac{2x_{5}(t)}{\tau_{e}}\\ {\dot{x}}_{6}(t) & =\frac{g_{4}h_{i}}{\tau_{i}}\left(\frac{1}{\exp^{-0.56x_{7}(t-\delta )}\;+1}-0.5\right)-\frac{x_{3}(t)}{\tau_{i}^{2}}-\frac{2x_{6}(t)}{\tau_{i}}\\ {\dot{x}}_{7}(t) & =x_{8}(t)\\ {\dot{x}}_{8}(t) & =\frac{g_{3}h_{e}}{\tau_{e}}\left(\frac{1}{\exp^{-0.56x_{9}(t-\delta )}\;+1}-0.5\right)-\frac{x_{7}(t)}{\tau_{e}^{2}}-\frac{2x_{8}(t)}{\tau_{e}}\\ {\dot{x}}_{9}(t) & =x_{5}(t)-x_{6}(t). \end{array} & \end{array}$$

**Table 1.** Parameters in a DCM of ERPs [39] and their description. True values used to generate the data, along with the shape and scale of the Gamma prior placed for Bayesian analysis that ensure that about 50% of the sampled parameters result in unstable dynamics of the system [16,17] . EPSP: excitatory postsynaptic potentials; IPSP: inhibitory postsynaptic potentials.

parameter	description	value	prior shape	prior scale
$g_{1}$	connection pyramidal⟶stellate	0.42	18.16	0.03
$g_{2}$	connection stellate⟶pyramidal	0.76	29.9	0.02
$g_{3}$	connection pyramidal⟶inhibitory	0.15	29.14	0.005
$g_{4}$	connection inhibitory⟶pyramidal	0.16	30.77	0.007
δ	intrinsic delay	12.13	22.87	0.51
$\tau_{i}$	rate-constant of IPSP	7.77	34.67	0.23
$h_{i}$	maximum amplitude of IPSP	27.88	20.44	0.96
$\tau_{e}$	rate-constant of EPSP	5.77	33.02	0.16
$h_{e}$	maximum amplitude of EPSP	1.63	24.17	0.07
u	input current	3.94	23.62	0.13

We simulate observations of pyramidal neuron voltage by (Euler-) integrating the model with a time step of dt=0.1 ms (figure 1B). A zero-centred Gaussian noise with a standard deviation of 0.1 is added to the observations (N=2001), which are then downsampled by a factor of 10 (N=201).

### 2.3. Bayesian inference

Bayesian inference allows to take advantage of background knowledge about the parameters θ of a statistical model while updating this knowledge given new information provided by observed data y. The background knowledge, or prior, is encoded as a probability distribution p(θ), which reflects assumptions about the quantities to be inferred, before seeing the data. These assumptions are updated under the influence of the data, quantified by the likelihood p(y|θ), providing the posterior distribution p(θ|y), up to a normalizing constant,


(2.2) $$\begin{array}{lcr} & p(\theta |y)=\frac{p(\theta )p(y|\theta )}{p(y)}\propto p(\theta )p(y|\theta ). & \end{array}$$

In this work, our goal is to infer the parameters of a DCM of ERPs given a simulated response of pyramidal neurons (see equation (2.1) and figure 1). We assume a Gaussian likelihood and place Gamma priors parametrized according to table 1 on model parameters.

### 2.4. Hamiltonian Monte Carlo

MCMC [53] methods are the gold-standard for generating samples from the posterior distribution by constructing a Markov chain that converges to this target distribution. HMC [22,54] is the state-of-the-art MCMC algorithm that leverages deterministic Hamiltonian dynamics to inform Markov chains, enabling more efficient exploration of the target distribution. HMC introduces an auxiliary momentum parameter ρ to be jointly evolved with the position of model parameters θ in the parameter space, by integrating the Hamiltonian equations of motion given by


(2.3) $$\begin{array}{rl} \frac{d\theta }{dt} & =\frac{\partial H}{\partial \rho }\\ \frac{d\rho }{dt} & =-\frac{\partial H}{\partial \theta }, \end{array}$$

where H(θ,ρ)≡−log⁡π(θ,ρ)≡−log⁡π(ρ|θ)−log⁡π(θ)≡K(ρ,θ)+V(θ) is the Hamiltonian function. In this representation, K(ρ,θ) accounts for the kinetic energy and V(θ) for the potential energy. The leapfrog integrator intertwines half updates of momentum with full update of model parameters,


(2.4) $$\begin{array}{rl} & \rho_{n+\frac{1}{2}}\leftarrow \rho_{n}-\frac{\epsilon }{2}\frac{\partial V}{\partial \theta }(\theta_{n})\\ & \theta_{n+1}\leftarrow \theta_{n}+\epsilon \rho_{n+\frac{1}{2}}\\ & \rho_{n+1}\leftarrow \rho_{n+\frac{1}{2}}-\frac{\epsilon }{2}\frac{\partial V}{\partial \theta }(\theta_{n+1}), \end{array}$$

where ϵ is the step size. Outputs of the integration are then subjected to an accept–reject step [55,56]. Hence, it is critical to monitor divergence, as it indicates that the numerical simulation of the Hamiltonian dynamics has failed, typically due to an energy increase suggesting a step size that is too large for complex geometries [57–59].

Although superior to gradient-free sampling algorithms, the performance of HMC is highly sensitive to the step size and the number of steps in the leapfrog integrator used to update the position and momentum variables in Hamiltonian dynamic simulation [23,57,59]. Its adaptive variant, the NUTS [23], implements a recursive path to dynamically tune on-the-spot the hyperparameters (step size and number of steps) of the symplectic integrator, thereby eliminating the need for hand tuning of these algorithmic parameters. For instance, the accept rate parameter (between 0 and 1) is used to control the target acceptance probability for proposed steps during sampling, while the tree depth parameter controls the maximum number of leapfrog steps (specified as $2^{N}$).

The self-tuning sampler automatically adjusts trajectory length based on the behaviour of the target distribution. However, due to numerical instabilities or highly curved and complex posterior geometries, the sampler may attempt to construct an infinitely long trajectory. To prevent this, a maximum trajectory length is imposed by setting the maximum tree depth parameter. Hamiltonian trajectories are numerically approximated by the leapfrog integrator, where the precision of this approximation is governed by the step size: for a fix length, a smaller step size results in more points along the numerical trajectory, thereby closely approximating the continuous trajectory, but at the expense of higher computational cost. Hence, the performance of HMC is contingent on the length of these trajectories. If the trajectory is too short, the sampler explores the parameter space inefficiently. On the other hand, if the trajectory is excessively long, the sampler may loop back to its starting point, leading to redundant exploration and the waste of computational time. Models with well-behaved geometry typically operate well with default hyperparameter values. However, for more complex models with intricate degeneracies, setting an appropriate proposal (or maximum) value for these hyperparameters necessitates careful consideration of the target distribution’s geometry to balance computational cost and accuracy [23,57–59].

### 2.5. Variational inference

VI [9,60] is an alternative to MCMC methods that approximates probability distributions through an optimization problem, which typically results in much faster computation than MCMC methods. The core idea of VI is to posit a fixed-form (i.e. a parametrized) family of distributions and then to find the member of that family which is close to the target [8,61,62]. In VI, closeness is expressed in the sense of the Kullback–Leibler (KL) divergence, which is an (asymmetric and non-negative) information-theoretic measure of proximity between two probability distributions. The goal is to minimize the KL divergence between the parametrized approximate posterior q(θ)∈Q from a family of tractable distributions and the true posterior p(θ|y). However, evaluating this divergence requires computing the evidence log⁡p(y), which becomes intractable. Instead, VI optimizes an alternative objective, which (by the fact that KL(.)≥0, or through Jensen’s inequality) forms a lower bound for log⁡p(y), the marginal log likelihood of the observed data, known as the evidence lower bound (ELBO; [63,64]) or negative free-energy [11,65],


(2.5) $$\begin{array}{rl} \text{ELBO}(q)\; & =E_{q(\theta )}[\log p(\theta ,y)]-E_{q(\theta )}[\log q(\theta )]\\ & =\log p(y)-\text{KL}\left[q(\theta )||(p(\theta |y)\right]\\ & \leq \log p(y). \end{array}$$

This allows us to perform approximate Bayesian inference by maximizing the ELBO with respect to q(θ), instead of directly minimizing the intractable KL divergence between the variational distribution and the true posterior.

In practice, VI transforms an inference problem into an optimization problem, requiring a full pass through the data at each iteration [56]. Stochastic variational inference (SVI; [13]) defines local and global variational parameters and uses natural gradients in a stochastic optimization algorithm over subsamples (minibatches) of data to enhance the speed and scalability of variational approaches. In this study, we opted for SVI using NumPyro [29], a probabilistic programming library designed for fast, flexible and scalable Bayesian modelling and inference. In addition to user-defined variational families, NumPyro supports several automatic guides that derive variational families from the generative model [66,67]. Guides available in NumPyro include the mean-field and full-rank Gaussians, low-rank Gaussian and variational distributions parametrized by normalizing flows (such as inverse autoregressive flow [68] and block neural autoregressive flow [69]) that rely on the autoregressive property and transformations by neural networks to convert a simple distribution to any target.

In addition, NumPyro offers an automatic Laplace approximation. Laplace’s method approximates a function near its mode as a Gaussian density by leveraging its second-order Taylor expansion (quadratic approximation). The mean (mode) of the target Gaussian distribution is determined by the argmax of the function, while the covariance matrix is computed as the inverse of the negative Hessian matrix evaluated at the mode. Hence, the Laplace approach relies on the analytical solution, but to make it feasible, the log posterior needs to be twice-differentiable. Laplace variational inference, as the primary approach used in DCM [10–12] leverages a quadratic approximation to the variational energy log⁡p(y,θ) and defines a proposal quadratic approximated posterior $q(\theta )\equiv N(\mu ,\Sigma^{-1})$, where


$$\mu ={\text{argmax}}_{\theta }\log p(y,\theta ),\;\;\;\;\;\;\;\;\;\;\;\Sigma =-\frac{\partial^{2}}{\partial \theta^{2}}\log p(y,\theta ){|}_{\mu }$$

and gives the following expression for the ELBO under the Laplace approximation:


(2.6) $${\text{ELBO}}_{\text{Laplace}}=\log p(y,\mu )+\frac{1}{2}\log|\Sigma |+\frac{k}{2}\log2\pi +\frac{k}{2},$$

where k is the number of parameters and |⋅| denotes the determinant. In NumPyro’s implementation of variational Laplace in unconstrained space, a point estimate is inferred (maximum *a posteriori*, i.e. ${\text{argmax}}_{\theta }\log\;p(\theta |y)$, which is equivalent to ${\text{argmax}}_{\theta }\log\;p(y,\theta )$) for the mean μ, by using Dirac delta distributions as (intermediate) variational family. The inverse of the Hessian is evaluated at this point, using JAX’s auto-differentiation for computing the Hessian. JAX [30,70] is a Python library that provides a high-level tracer for implementing transformations (e.g. automatic differentiation, vectorization and just-in-time (JIT) compilation) of Python functions. NumPyro wraps several JAX optimizers to be used in SVI. In this work, we use Adam [71] with learning rate 0.0005.

Nevertheless, the model-specific derivation of VI requires a tedious and extensive effort to define a variational family appropriate for the probabilistic model, compute the corresponding objective function and gradients, and execute a gradient-based optimization algorithm. Automatic differentiation variational inference (ADVI; [72]) solves this problem automatically, in a black-box manner [73], without requiring analytic derivation or manual tuning by the user. ADVI is an automatic implementation of VI that derives an efficient and generic variational inference solution, requiring only a statistical model and dataset, without the need to specify a variational family or rely on conjugacy assumptions [72]. ADVI employs an unconstrained real-coordinate space mapping θ↦ζ that facilitates the use of a single variational family for all models in a large class, implicitly generating non-Gaussian variational distributions in the original space. The mean-field implementation posits a factorized Gaussian variational approximation, $q(\zeta )\equiv \prod_{k=1}^{K}N\left(\mu_{k},\sigma_{k}^{2}\right)$, that is a product of Gaussians, with variational parameters $\{\mu_{k},\sigma_{k}^{2}{\}}_{k}$, ignoring off-diagonal terms of the covariance (i.e. the degeneracy between parameters). The full-rank variant defines $q(\zeta )\equiv N\left(\mu ,LL^{T}\right)$ with variational parameters ϕ={μ,L} to be optimized, thereby capturing posterior correlations and producing more accurate estimates of marginal variances. In ADVI, the ELBO objective is approximated using Monte Carlo integration, and is to be maximized with stochastic (automatic differentiation-) gradient-based optimization (specifically, AdaGrad; [74]). In this work, we use the ADVI implementation [72] in the probabilistic software Stan [27].

### 2.6. Probabilistic programming languages

PPLs [25] are a paradigm for automatic statistical modelling and machine learning that allows for the definition, manipulation and inference of complex probabilistic models using high-level programming languages. They enable users to specify probabilistic models in a more intuitive and flexible manner, while leveraging high-performance computing and automatic differentiation (AD) through dependencies on specialized libraries. Central to the functionality of many PPLs is the concept of AD, a computational technique that efficiently and accurately calculates the derivatives of functions [24]. Unlike symbolic differentiation, which involves manipulating mathematical expressions, or numerical differentiation, which approximates derivatives using finite differences, AD breaks down functions into elementary operations and systematically applies the chain rule to compute derivatives. AD computes derivatives to machine precision, thereby avoiding the approximation errors inherent in numerical differentiation. It is notably efficient, with the computational complexity of obtaining gradients comparable with that of evaluating the function itself. AD plays a crucial role in enabling efficient and accurate inference using gradient-based methods in PPLs.

As of today, numerous Bayesian analysis software and packages offer embedded automatic differentiation and probabilistic sampling. While Stan has long been established in the statistical community, the rise of machine learning in the last decade has considerably boosted the development of automatic differentiation packages, paving the way for numerous gradient-based inference tools to be efficiently accessible. Although, historically, statisticians have shown a penchant for the R programming language (for example, the R interface for Stan RStan was created in 2013, the Python interface PyStan in 2018), the grown popularity of Python within the scientific community has boosted the development of inference packages in this language. Pyro [75] is a universal PPL based on PyTorch [76]. NumPyro [29] is a lightweight version of Pyro that runs on the JAX framework [30,70], for efficient program optimization, parallelization and GPU/TPU acceleration.

In this study, we implemented inference on a DCM of ERPs (see equation (2.1)) in several open-source PPLs from the top 20 most-downloaded packages related to Bayesian inference (see [26], which also provides the list of R packages): CmdStanPy (Python interface to CmdStan [77]), PyMC [28], NumPyro [29] and BlackJAX [78]. Although the choice of which PPL to use is at the convenience of the user, we benchmarked their efficiency, with a focus on the NUTS algorithm.

### 2.7. Model comparison and stacking

Model comparison involves evaluating and comparing different statistical models to determine which one best explains the observed data, taking into account both accuracy and complexity [79]. The criteria to assess and compare models balance model evidence, (or likelihood), that is, the fit to the data, and a penalty for model complexity, thus favouring models that achieve a trade-off between simplicity and explanatory power. Simplistic criteria like the Akaike information criterion (AIC; [80], given by equation (7)) or its Bayesian variant (BIC; [81], given by equation (2.8)) are well-known and used according to a point estimation [82,83],


(2.7) $$\text{AIC}=-2\log p(y|{\hat{\theta }}_{\text{mle}})+2k$$

(2.8) $$\text{BIC}=-2\log p(y|{\hat{\theta }}_{\text{mle}})+k\log(n),$$

where $\log\;p(y|{\hat{\theta }}_{\text{mle}})$ is the maximum log likelihood, k is the number of parameters in the statistical model and n is the number of data points.

In the Bayesian context, where we have access to the full posterior distribution, other criteria have been proven to be more suitable [32,84]. Based on expected log pointwise predictive density (elpd), the approximate Watanabe–Akaike information criterion (WAIC; [85–87], given by equation (2.9)) and cross-validated leave-one-out (CV-LOO; [84,85], given by equation (2.12)), provide a more nuanced assessment of model fit and complexity.

The formula to compute the WAIC criterion is given by [84]


(2.9) $$\begin{array}{lcr} & {\hat{\text{elpd}}}_{\text{waic}}=-2\left(\hat{\text{lpd}}-{\hat{p}}_{\text{waic}}\right) & \end{array}$$

with the computed log pointwise predictive density $\hat{\text{lpd}}$ based on averaging with respect to posterior samples $(\theta_{s}{)}_{s=1}^{S}$, and simulation-estimated effective number of parameters ${\hat{p}}_{\text{waic}}$, computed from the empirical variance given posterior samples,


(2.10) $$\hat{\text{lpd}}=\sum_{i=1}^{n}\log\left(\frac{1}{S}\sum_{s=1}^{S}p(y_{i}|\theta_{s})\right)$$

(2.11) $${\hat{p}}_{\text{waic}}=\sum_{i=1}^{n}{\hat{\text{Var}}}_{s=1}^{S}\log p(y_{i}|\theta_{s}).$$

The Pareto-smoothed importance sampling approximation of CV-LOO (denoted by PSIS-LOO), which eliminates the need to perform actual leave-one-out cross-validation, is given by


(2.12) $${\hat{\text{elpd}}}_{\text{psis-loo}}=-2\sum_{i=1}^{n}\log\left(\frac{\sum_{s=1}^{S}w_{i}^{s}p(y_{i}|\theta^{s})}{\sum_{s=1}^{S}w_{i}^{s}}\right),$$

where $(w_{i}^{s}{)}_{s=1}^{S}$ are Pareto-smoothed importance sampling weights [85].

In the DCM framework, the most popular inference algorithm, (Laplace) VI, is based on maximizing the ELBO (given by equation (2.5)), also known as the negative free-energy [11]. This approach is widely used for model selection in DCM [31].

Related to model comparison, *stacking* to average Bayesian predictive distributions [38,88], weights and combines the distributions from multiple models (or chains) to provide more robust inference and enhance predictive performance. Given K models $M_{1},\ldots M_{K}$, the weights computed based on stacking of predictive distributions, are the solution of the optimization problem


(2.13) $$\max_{w}\frac{1}{n}\sum_{i=1}^{n}\log\sum_{k=1}^{K}w_{k}p\left(y_{i}|y_{-i},M_{k}\right),\;\text{ s.t. }\;w_{k}\geq 0,\sum_{k=1}^{K}w_{k}=1,$$

where $y_{-i}$ is data without the ith data point and the leave-one-out predictive density $p(y_{i}|y_{-i},M_{k})$ can be estimated with ${\hat{\text{elpd}}}_{\text{psis-loo}}$ (equation (2.12)). Thanks to this approximation, readily implemented in the ArviZ package [89], the computation of stacking weights is fast, negligible compared with that of the inference part. We use stacking of predictive distributions to address post hoc the discrepancies and multi-modality observed when running multiple chains or repetitions of inference on an ill-defined model. It is worth noting that the stacking approach is not limited to outputs of MCMC algorithms, and can be applied as well to VI [38,88].

### 2.8. Convergence diagnostics

After running an MCMC sampling algorithm, it is necessary to conduct statistical analysis to evaluate the convergence of the chains. The Gelman–Rubin statistic $\hat{R}$ [90], which approaches 1 under optimal convergence, is the standard estimator to verify the within- and between-mixing of multiple Markov chains. Effective sample size (ESS; [91]) is another useful indicator that quantifies the uncertainty relative to the estimation of posterior quantities, such as quantiles, from the typically auto-correlated draws from MCMC [92]. A strong sampling efficiency in the core of the posterior is characterized by a high bulk ESS, which is computed for rank normalized values using split chains [91]. The sampling efficiency in the tails of the posterior is defined as the minimum of the ESS for the 5% and 95% quantiles.

More generally, in Bayesian analysis, useful quantities for detecting issues in inference include posterior *z*-scores, computed for each model parameter, which are a combination of bias and precision of the posterior, $z=\frac{E(p(\theta_{i}|y))-\theta_{i,true}}{std(p(\theta_{i}|y))}$, and should be close to 0 in the ideal case [57,93].

### 2.9. Hardware set‐up

All experiments were conducted on a Linux machine with a 3.60 GHz Intel Core i7-7700 CPU (four cores) and 32 GB of memory.

## 3. Results

In this section, we present our findings on various strategies and comparisons aimed at improving and evaluating Bayesian inference methods for a DCM of ERPs (see equation (2.1) and figure 1). We begin by outlining three strategies for addressing multi-modality in inference: fine-tuning the hyperparameters of the sampler, leveraging initialization and employing stacking techniques based on predictive accuracy. Following this, we provide a comparison between VI family and NUTS in fitting the ODE model, highlighting the strengths and limitations of each approach. We then conduct Bayesian model comparison under both variational and MCMC frameworks, ensuring consistency in model evaluation. Lastly, we benchmark the sampling efficiency of three popular PPLs (namely, NumPyro, PyMC and Stan), showcasing the impact of programming environment on inference performance.

### 3.1. Multi-modality

Multi-modality is often encountered in biological systems [34], when distinct sets of parameter values can explain the data equally well. In MCMC, running multiple Markov chains with diverse starting points is a standard practice to ensure the reliability of inference. This approach helps in assessing convergence and ensures that the chains have explored different regions of the parameter space. By comparing the results of multiple chains, one can determine if they are converging to the same region or if there are significant discrepancies indicating multiple modes. It can also happen that multi-modality is not present in the true data generating process, but rather is a result of the inference process. When the fit to the data or the posterior predictive checks are inadequate, it indicates issues such as a misspecified model, data quality concerns or problems with the inference method. Regarding the latter, ensuring that the algorithm used to estimate the posterior does not get stuck near a local optimum is crucial.

**Figure 2.**
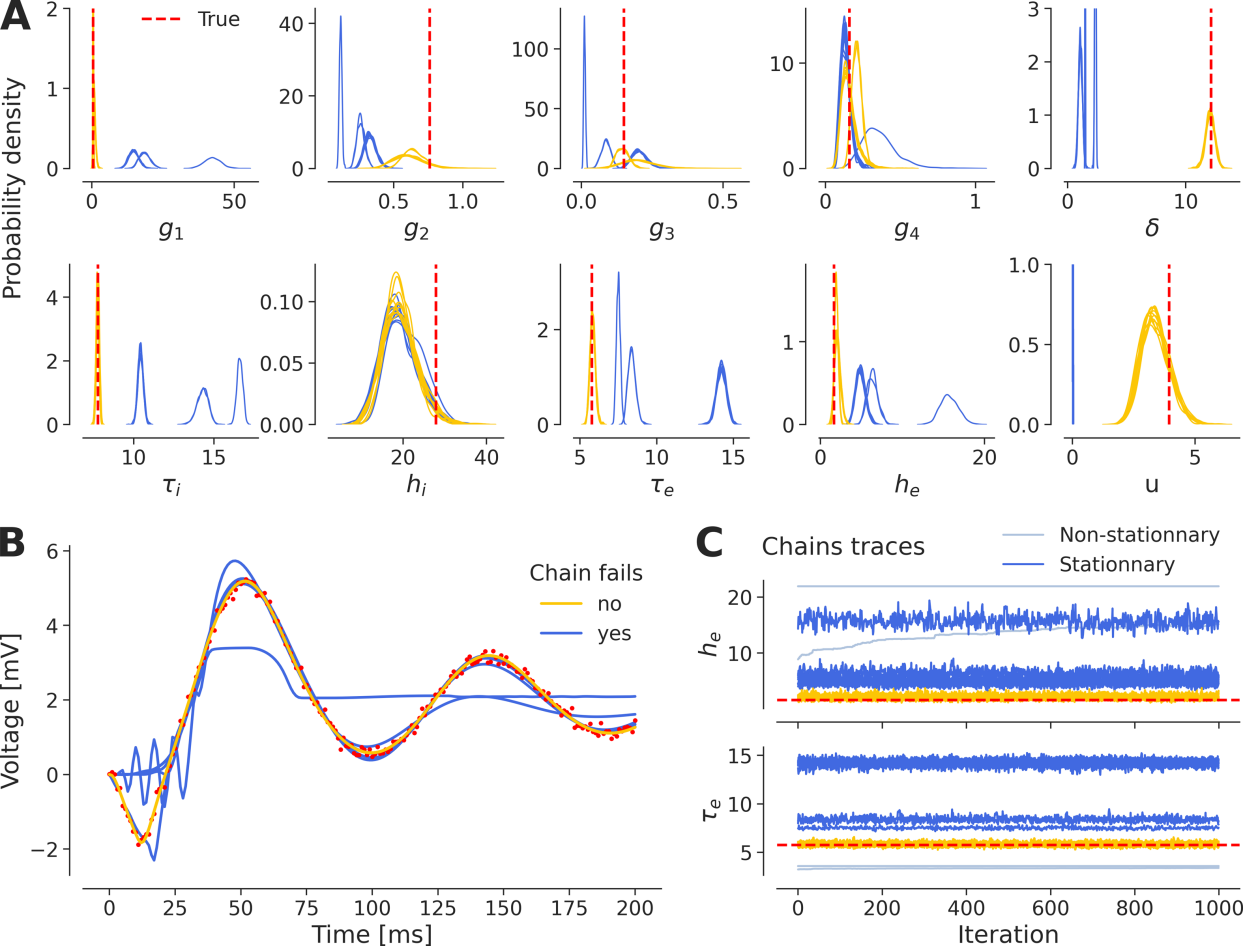
Multi-modality arises in the inference process. (A) The estimated posterior distributions (24 chains), coloured depending on their goodness-of-fit (in blue and yellow, as in B and C) are targeting different regions or modes of the parameter space. True values are shown as a dashed red line. (B) Chains that fall into local optima (in blue) in the parameter space fail also to recover perfectly the true dynamics of the system (in red), contrary to the chains that target the correct posterior mode (in yellow). (C) Chains that show non-stationary patterns are discarded (in light blue); however, a chain can converge to a stationary distribution that is a local solution (in blue) rather than the global optimum (in yellow).

We encounter discrepancies in the results of repeated inference (e.g. multiple HMC chains) on the DCM of ERPs (see figure 1 for the generated observations). We perform a first round of inference on the NMM model (equation (2.1)) for which we set diffuse Gamma priors (see electronic supplementary material, table S1), illustrating the multi-modality that arises during the inference process (figure 2A). The 24 chains, ran with random initial conditions, can fail to recover the system dynamics (figure 2B), and are therefore not indicative of true multi-modality in the data generating process, rather they are misleading the inference. Nevertheless there could exist other modes capable of generating accurate dynamics in observation (see electronic supplementary material, figure S1). The chains that show non-stationary patterns are discarded; however, several chains show convergence to a stationary distribution that nevertheless does not reflect the true data generating distribution (figure 2C). These results indicate the challenges in achieving convergence to the true data generating distribution, especially given a high-dimensional ODE model and diffuse priors.

Placing an informative prior has been proposed as a solution to mitigate the issues of multi-modality and degeneracy [32,94]. In the rest of this paper, we use a set of weakly informative Gamma priors motivated by [16,17] as described in table 1. We note that enforcing even more informative priors does not guarantee to solve the issues of multi-modality, as we have shown in electronic supplementary material, figure S2, and we need to find other solutions to address the exploration issues encountered by the inference scheme. It is important to note that this lack of convergence is not a flaw specific to MCMC sampling (here, NUTS) and using other methods such as variational inference result in the same issue (see electronic supplementary material, figure S3).

**Figure 3.**
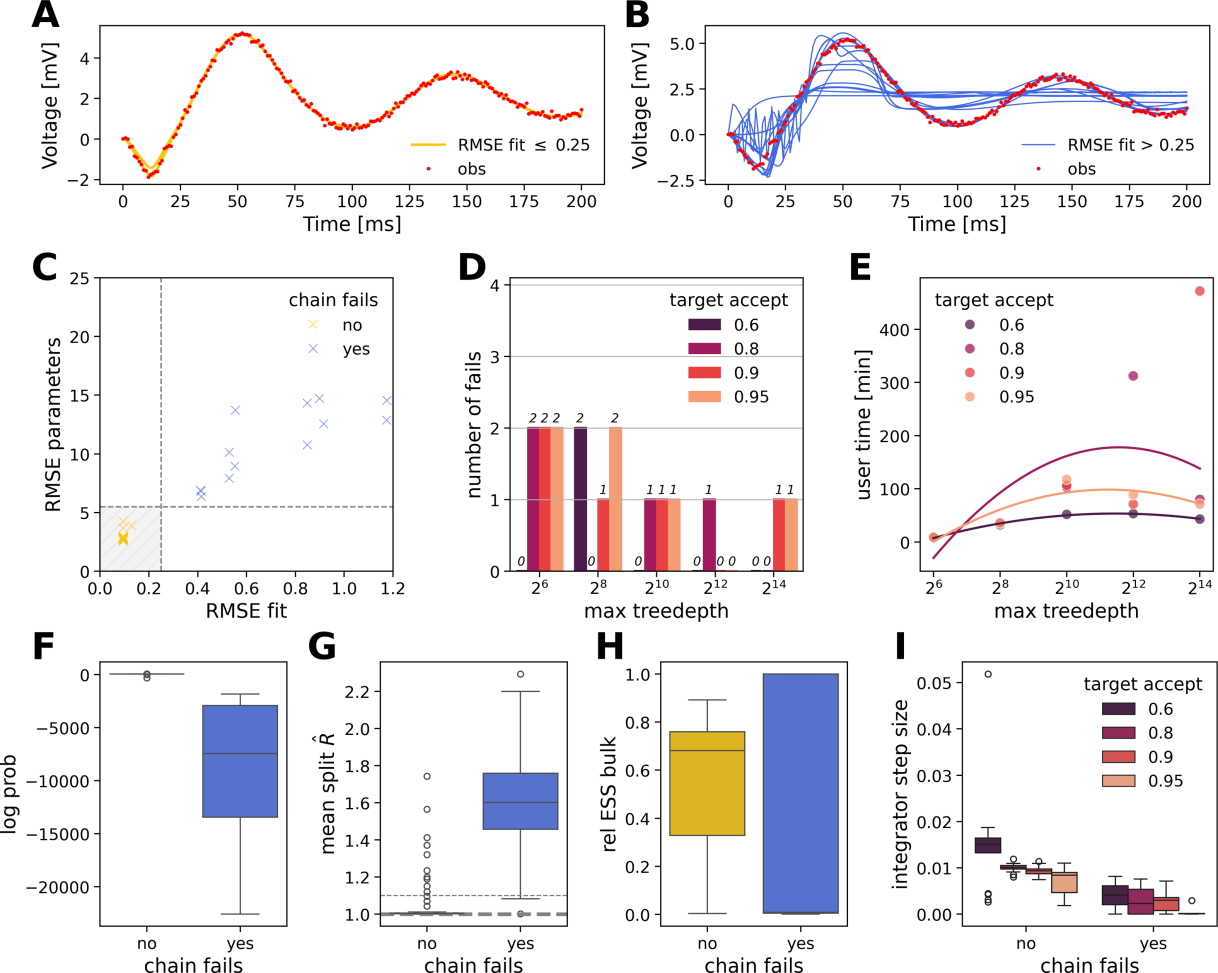
Fine-tuning hyperparameters of the No-U-Turn Sampler. (A) Observations (in red) and faithful fits (RMSE≤0.25, in yellow). (B) Observations (in red) and inaccurate fits (RMSE>0.25, in blue). (C) Estimation error in retrieving the model parameters (RMSE parameters) versus the estimation error in retrieving the observation (RMSE fit). There is a clear relationship between these two quantities and successful inferences (in yellow) are located within the shaded grey area. (D) Count of chains that fail to recover the data, out of four parallel chains, with respect to 20 grid-searched NUTS hyperparameter settings. (E) Computational time of NUTS (with 1000 warm‐up and 1000 samples) with respect to hyperparameter settings. Second-order polynomial regression fit curves are given as reference. (F) log probability, (G) mean split $\hat{R}$, computed with the two halves of a chain as a measure of within-chain convergence and (H) relative effective sample size (ESS) in the bulk of the posterior. (I) Step-size of the leapfrog integrator with respect to target acceptance rate.

As the first step, we aim to improve the convergence of the algorithm by enhancing the performance of the integrator (see figure 3). Adjusting the hyperparameters of the inference algorithm, such as the step size and the number of leapfrog steps in HMC, may improve exploration in the search space. Adaptive versions of these algorithms, such as NUTS, can automatically tune these parameters during the warm-up phase. However in NUTS, other important parameters include the maximum tree depth of the binary tree representing the evolution of the integrator, ensuring finite-time integration if the No-U-Turn termination criterion is never met. Additionally, the target acceptance rate of Hamiltonian trajectories submitted in a Metropolis step influences the integration step size.

Here we demonstrate the results of fine-tuning the hyperparameters of NUTS to ensure both within-chain and between-chain convergence. In figure 3, we summarize the inference results obtained for 20 combinations (grid-search) of hyperparameters, in terms of fit to the data (RMSE fit), closeness of estimated parameters to the ground truth (RMSE parameters), log probability (as a measure of prediction for the observed data) and convergence indicators, such as relative ESS and split $\hat{R}$ (see §2).

Figure 3A,B illustrates the observations and successful overlapping fits (figure 3A, with RMSE≤0.25), or failed recoveries of the observation (figure 3B, with RMSE>0.25), out of a total of 80 chains (20 different hyperparameters configurations, run with four chains each). From figure 3C, we observe that a poor fit to the data is closely related to a poor estimation of model parameters. In figure 3D, we reported the number of chains that fail to recover the data and ground truth parameters, for each tested pair of hyperparameters (target acceptance and max tree depth). For this specific model and data, a tree depth of $2^{8}$ nodes is sufficient to prevent premature termination of Hamiltonian integration but is not a guarantee of sufficient exploration that achieves convergence. Setting the target acceptance rate to 0.6 solved the convergence issues (figure 3D).

In the face of challenging convergence, the computation time does not follow a strict law and heavily depends on a chain being stuck in a local minimum (figure 3E). Chains that are able to faithfully fit the data show a significantly higher log probability (figure 3F), as well as better within-chain mixing and convergence (see split $\hat{R}$; figure 3G). However, there is no clear difference in effective sample size (figure 3H), except that chains that fail in the task of recovering the data or ground truth parameters can achieve either extremely low or extremely high ESS. Setting a low target acceptance rate enforced exploration and enabled larger integration steps (figure 3I). It also shows that in this model, successful fits are characterized by enhanced exploration, with, on average, larger step sizes. These results indicate the critical effect of algorithmic parameters on efficient and accurate inference, while their hand-tuning for convergence is painstaking work and does not easily generalize.

**Figure 4.**
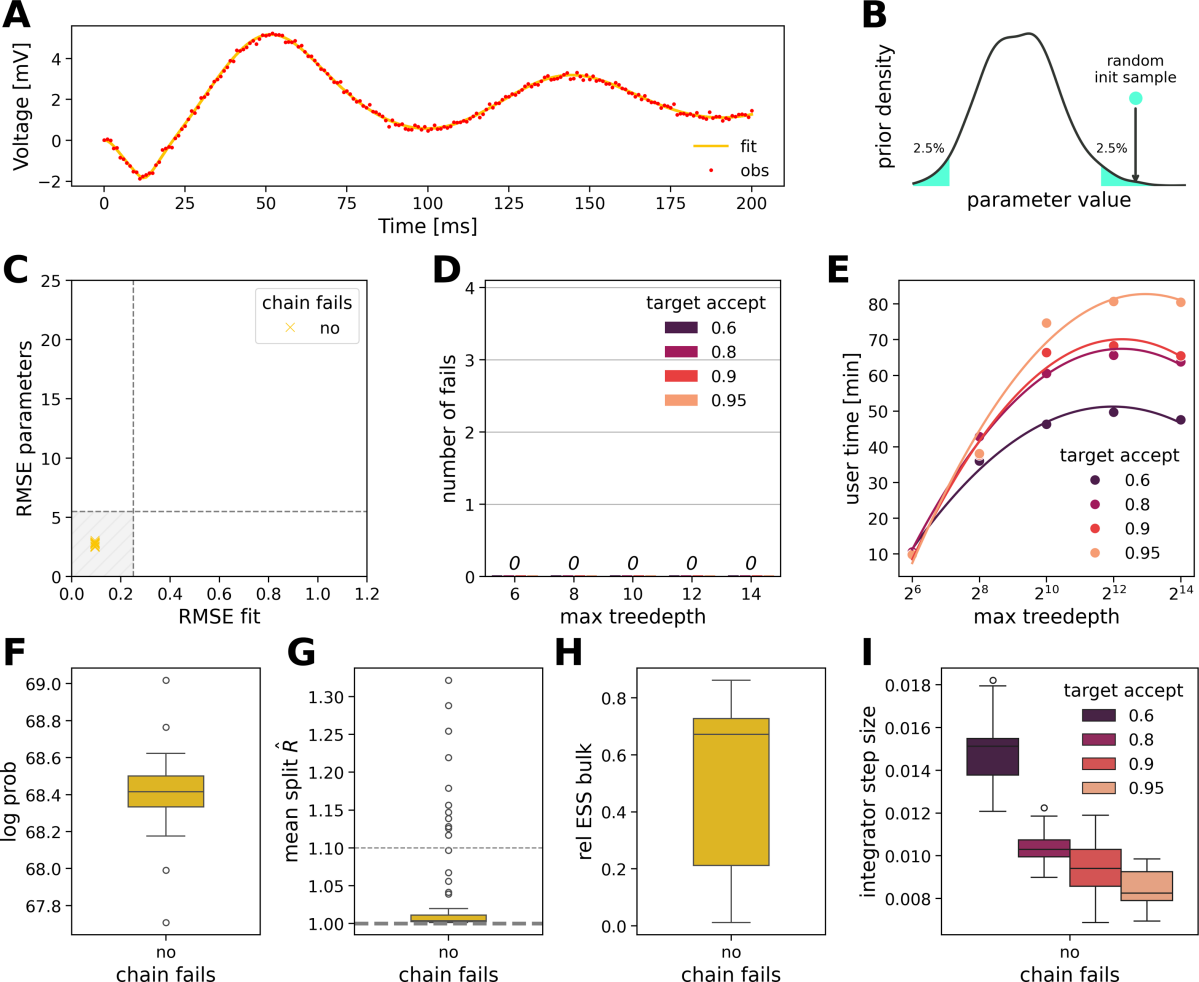
Initialization of the chains in the tails of the prior as a remedy for convergence issues. (A) All of the 80 chains successfully recover the observed data. (B) Initialization scheme: the initial position of a chain in parameter space is set to a random sample taken uniformly in the tails of the priors. (C) Estimation error in retrieving the model parameters (RMSE parameters) versus the estimation error in retrieving the observation (RMSE fit). All initialized runs are successful inferences and are located within the shaded grey area. (D) No initialized chain, out of a total 80 chains, fails to recover the data, run with 20 grid-searched NUTS hyperparameter settings. (E) Computational time of NUTS (with 1000 warm‐up and 1000 samples) with respect to hyperparameter settings. (F) Log probability, (G) mean split $\hat{R}$, computed with the two halves of a chain as a measure of within-chain convergence and (H) relative effective sample size (ESS) in the bulk of the posterior. (I) Step-size of the leapfrog integrator with respect to target acceptance rate.

As the second step, we aim to improve convergence by leveraging the information provided by the weakly informative priors (see table 1). An alternative to the tedious tuning of algorithmic parameters is initializing the chains in a non-zero probability region of the prior. Initializing chains with a random sample taken uniformly from the tails of the prior (specifically below the 2.5th and above the 97.5th percentile, constituting a 5% probability region) ensured 100% convergence, regardless of the values of HMC hyperparameters (but within reasonable ranges), as shown in figure 4. We prefer initialization in the tails rather than around the mode, as we believe this approach will generalize better to high-dimensional spaces where the target typical set does not include the region around the mode (see §4).

For the observation (figure 4A) and initialization strategy at the tails (figure 4B), we can see that in this case, all the chains converged successfully without any failures, out of a total of 80 chains (figure 4C,D). In such a regime where convergence is straightforward, the computation time scales quadratically with the maximum tree depth (figure 4E). The log probability (figure 4F), the split $\hat{R}$ (figure 4G) and the relative ESS (figure 4H) confirm the convergence of HMC chains. The influence of the target acceptance rate on the adaptation of the step-size of the leapfrog integrator is again highlighted in figure 4I. Large integrator steps are achieved (figure 4I), in agreement with the corresponding panel in the previous figure (figure 3I, where the chains that did not fail demonstrated a larger step-size on average compared with the ones that failed), favouring exploration through proposals over exact integration. These results demonstrate that initializing the chains at the tail of the prior provides a very straightforward and efficient convergence for our DCM model.

As the third step, we aim to propose a more generic solution for achieving chain convergence without manual tuning of algorithmic parameters or relying on the priors. This solution involves running standard practice randomly initialized chains, and subsequently stacking them post hoc [38,88]. This is achieved through weighted averaging based on their respective predictive power (see equation (2.13)).

**Figure 5.**
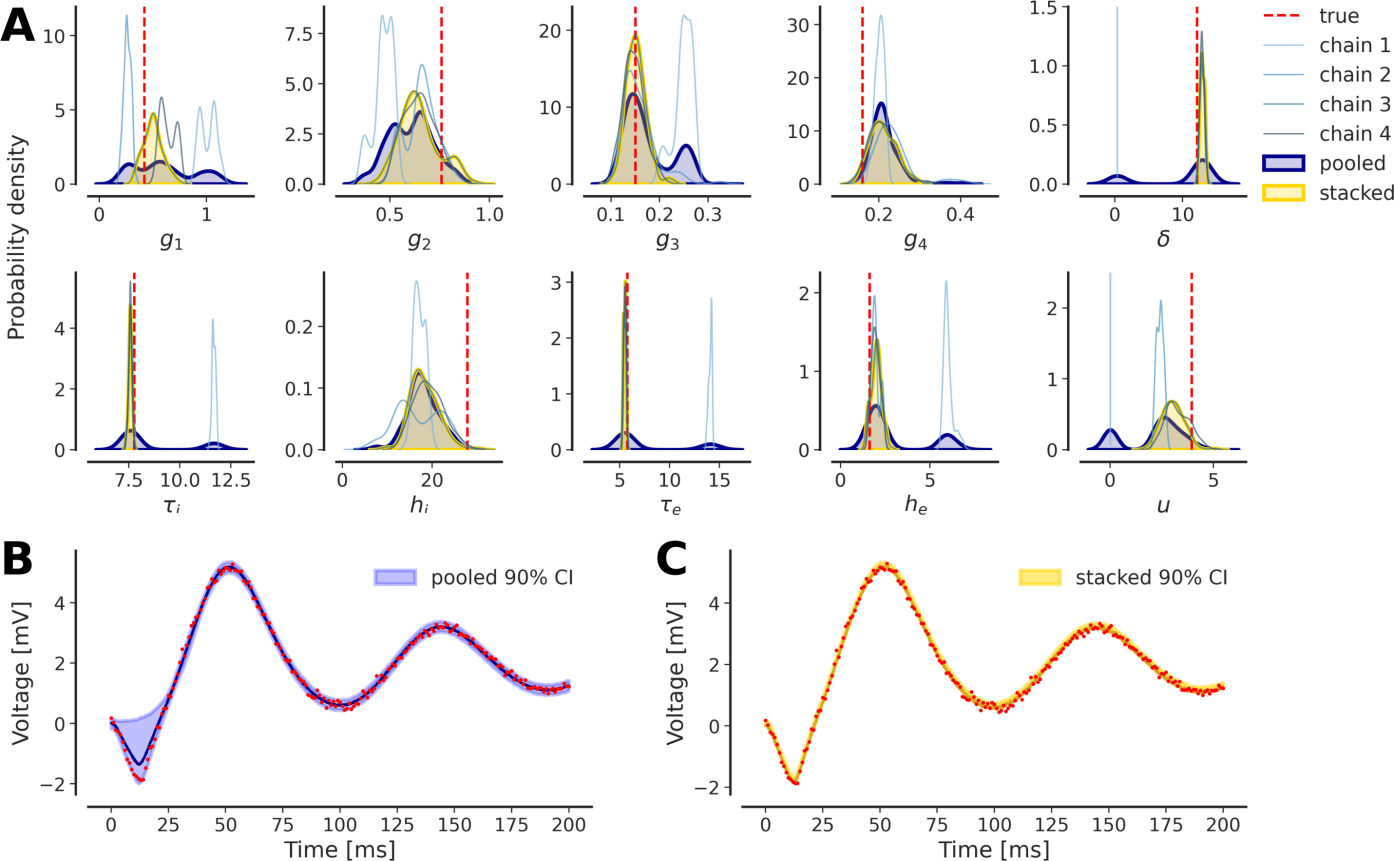
Addressing multi-modality in inference with stacking. (A) Markov chains (in shades of blue) have converged to different distributions that arise from different modes in the parameter space. (B) Pooling the chains (in dark blue) does not filter out artefact modes that do not perfectly reproduce system dynamics and data. (C) Stacking (in yellow) applies weights based on the predictive distribution of each chain and successfully smooths out local artefacts, resulting in better predictive performance.

In figure 5, we illustrate an example of mismatched inference where chains end up in different regions of the parameter space, some exhibiting Dirac delta-type distributions (i.e. strongly centred around a single point in the parameter space, indicating very low variability in the estimates of parameters). We can see that, unlike the naive pooling approach, which would consist of pooling all chains with equal weights, regardless of their respective predictive power, stacking the chains successfully smooths out local artefacts and phantom modes (figure 5A). Stacking to average Bayesian predictive distributions resulted in a perfect reproduction of the dynamics in the data, unlike pooling, which still suffers from errors induced by failed chains (figure 5B,C, respectively). It is worth noting that the stacking approach can also be applied to outputs of variational inference with multiple initializations [38,88].

In sum, these results indicate that achieving reliable and robust Bayesian inference for ODE models requires understanding the intricacies of the model and its parameters, and careful monitoring of convergence, even when relying on automated and state-of-the-art algorithms. Although, at first glance, the model seemed to produce inconsistent results, we were able to stabilize inference using three methods: the tedious one—hand tuning the algorithm, the simplest yet very effective one—initialization in the prior, and the more general approach—stacking the chains.

### 3.2. Algorithmic benchmark

Here we compare the performance of HMC versus VI methods. More specifically, we compare NUTS and (mean-field and full-rank variants of) ADVI, as well as the Laplace approximation, with initialization at the tails of the prior to ensure convergence. For consistency, we ran four parallel chains with NUTS (200 warm‐up and 200 sampling iteration, maximum tree depth of 10 and acceptance rate of 0.8), and four repetitions of variational inference (with $10^{5}$ iterations and a single ELBO sample).

In terms of computational time, the four NUTS chains using Stan took approximately 52 min, compared with 4 min using NumPyro. The four repetitions of mean-field and full-rank ADVI using Stan took approximately 19.4 and 18 min, respectively, in total (see table 2 for exact values). Using NumPyro, they took 3.22 and 12.98 min, respectively. These results demonstrate 2−13 times faster computational time using NumPyro for NUTS or ADVI. The automatic Laplace approximation proposed in NumPyro was the fastest of all methods, taking only 3.12 min total for the four repetitions of $10^{5}$ iterations. Hence, using NUTS or Laplace in NumPyro, we can sample the posterior in 3−4 min.

**Table 2.** Benchmarking of self-tuning variant of HMC, the No-U-Turn Sampler (NUTS), versus variational inference methods in solving an ODE model given by equation (2.1), using Stan and NumPyro implementations.

software	algorithm	time (s)	mean $\hat{R}$	RMSE fit	min. corr.	max. corr.	min. *z*-score	max. *z*-score
Stan	mean-field	1164.259	1.221	0.104	0.002	0.738	0.957	69.561
	full-rank	1080.777	1.006	0.104	0.015	0.985	0.115	1.260
	HMC-NUTS	3115.238	1.007	0.104	0.003	0.981	0.006	2.374
NumPyro	mean-field	193.583	1.062	0.009	0.004	0.234	1.067	266.635
	full-rank	779.127	1.003	0.097	0.002	0.922	0.422	19.579
	Laplace	187.758	1.002	0.097	0.004	0.982	0.150	2.475
	HMC-NUTS	245.1952	1.009	0.097	0.002	0.982	0.243	2.560

**Figure 6.**
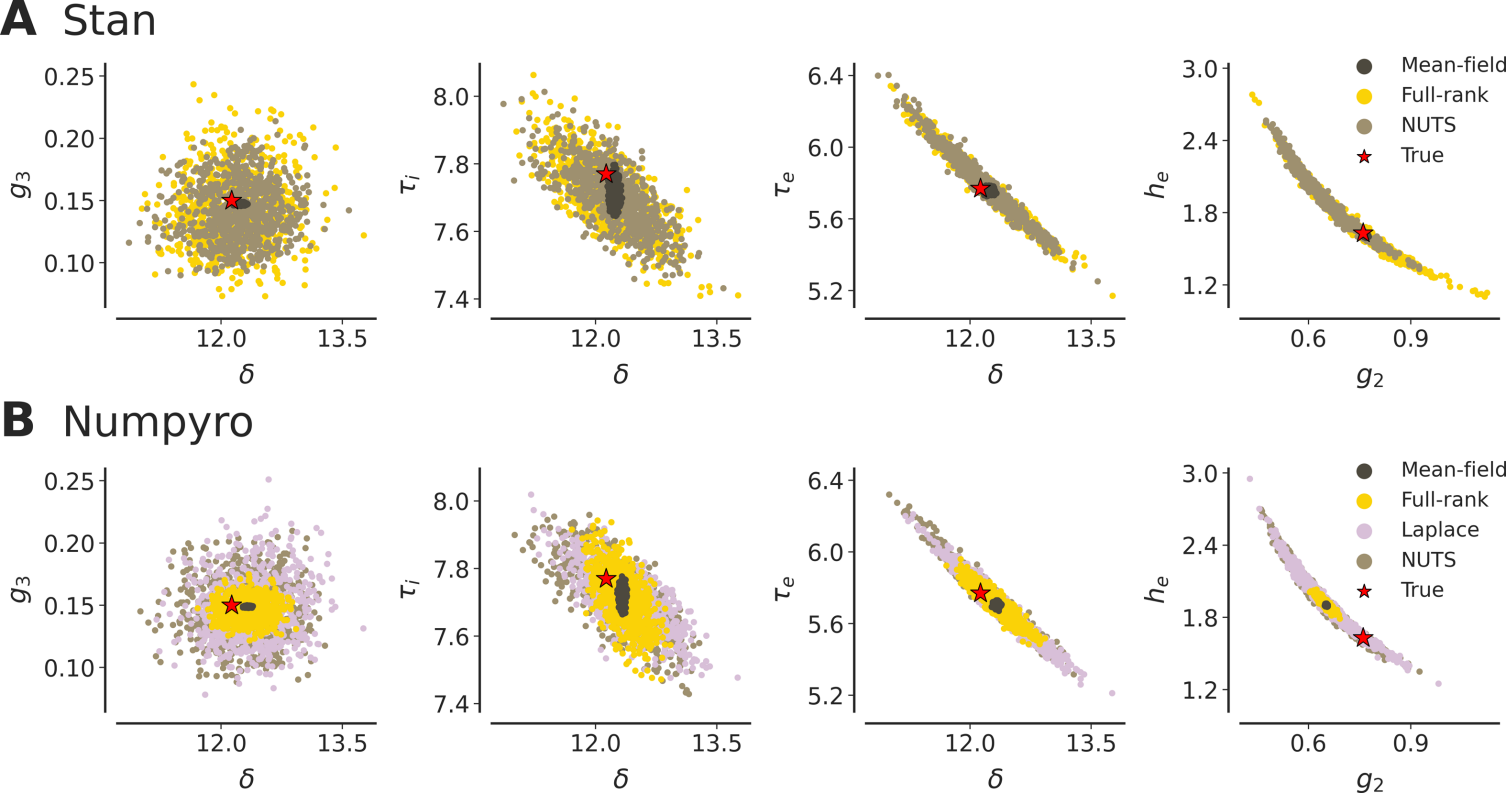
Pairplots of posterior samples obtained with variational inference methods (mean-field ADVI: dark brown, full-rank ADVI: yellow, automatic Laplace approximation: mauve) and HMC sampling (NUTS: light brown), for a selected subset of model parameters, using (A) Stan and (B) NumPyro. NUTS and the Laplace approximation both accurately provide posterior samples that encompass the true generative parameters (in red) and capture complex geometries in the posterior. In both softwares, the mean-field method underestimates marginal variances and produces uncorrelated and under-dispersed outputs. Numpyro's full-rank VI provides overconfident posteriors which can overlook the true parameter value.

Figure 6 displays the posterior samples obtained by each algorithm for a few selected parameters (see electronic supplementary material, figures S4 and S5, for pairplot of all parameters). Indicators of convergence and accuracy, the mean $\hat{R}$ across all parameters (although only interpretable for HMC), RMSE of the fit, minimum and maximum pairwise correlations, and *z*-scores are reported along with computational time in table 2. The NUTS, full-rank ADVI and (NumPyro’s) Laplace approximation, all show satisfactory convergence.

In terms of accuracy, the results indicated that, as expected, (Stan’s) full-rank ADVI performs equivalently to NUTS in capturing complex posterior geometries (e.g. see the pairplot for $g_{2}$ and $h_{e}$ in figure 6) and the true generative parameters, although it produces slightly more diffuse posterior distributions. NumPyro’s implementation of the full-rank Gaussian variational family gave significantly less dispersed posteriors than its Stan counterpart, at times overconfidently overlooking ground truth parameters (see the pairplot for $g_{2}$, and $h_{e}$ in figure 6). The Laplace approximation offered by NumPyro yields estimated posterior distributions that are equivalent to those obtained using NUTS. A known limitation of mean-field ADVI is the systemic underestimation of marginal variances [72], which we also observe for the model under study, in both Stan and NumPyro implementations. The mean-field algorithm provides uncorrelated and under-dispersed outputs, which fail to capture several of the ground truth model parameters.

In sum, these results indicate the accuracy and efficiency of estimation using NUTS and the Laplace approximation (the latter implemented only in NumPyro), with both algorithms producing reliable posterior distributions in 3−4 min.

### 3.3. Model comparison

**Figure 7.**
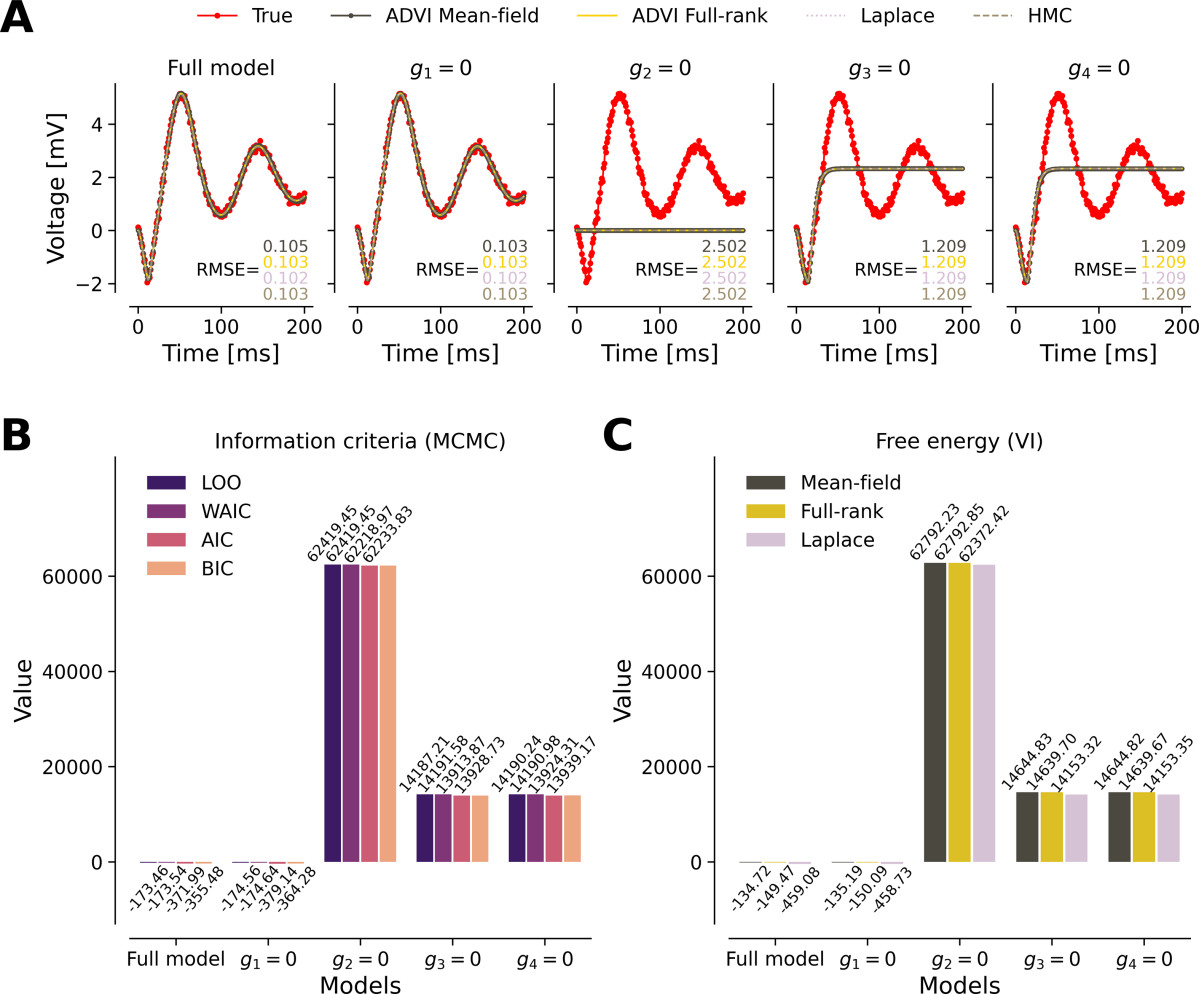
A complete taxonomy of model comparison methods. The full-model is compared with subset models that each set one connectivity parameter to zero. (A) Only the full model and the model where $g_{1}=0$ are able to recover the dynamics of the system. (B) Model comparison criteria (AIC, BIC, WAIC, LOO) for each model fitted with MCMC (here the NUTS algorithm). (C) Free-energy as a model comparison criterion in variational inference (here the mean-field and full-rank ADVI, and automatic Laplace algorithms). All criteria indicate that the reduced model with $g_{1}=0$ is superior, while the model with $g_{2}=0$ performs the worst. The smaller the value of criteria, the better the model's predictive ability.

This section focuses on model comparison, as a central tool within DCM. We generated observations using a full model of the system (see figure 1 and table 1). Subsequently, we fit an alternative model by removing connectivity between certain regions (see figure 7).

We observe that the full model and the model setting $g_{1}=0$—thus vanishing the strength of connection from pyramidal neurons to spiny-stellate cells—both perfectly fit the observation. However, setting one of $g_{2}=0$ (connection from spiny-stellate to pyramidal cells), $g_{3}=0$ (connection from pyramidal to inhibitory cells) or $g_{4}=0$ (connection from inhibitory to pyramidal cells) results in large deviation from the observation. More precisely, setting $g_{2}=0$ leads to a worse fit, whereas $g_{3}=0$ and $g_{4}=0$ result in similarly poor fits (see figure 7A, and the RMSE values).

We note that parameters for which estimation is most affected by the cuts in connections are the intrinsic delay δ, and rate constants of the membrane $\tau_{i}$ and $\tau_{e}$. Their posterior distribution estimated by ADVI and NUTS, with respect to each model, are shown with that of all other parameters in electronic supplementary material, figure S6.

Model comparisons based on classical AIC (equation (2.7)), and BIC (equation (2.8)) as well as Bayesian WAIC (equation (2.9)) and LOO cross-validation (equation (2.12)) in the MCMC framework (figure 7B) align closely with free-energy obtained from variational inference (figure 7C). Our model comparison results indicate that the model with $g_{1}=0$ is the best, while the model with $g_{2}=0$ performs the worst (figure 7B,C). Interestingly, setting parameter $g_{1}=0$ causes no loss in fitting the observation performance compared with the full model. Hence, the model comparison favours such a reduced model, as it diminishes by one the number of fitted parameters. Note that a smaller value of comparison criteria indicates a better model’s predictive ability.

Although this is straightforward in the definition of AIC/BIC, as the penalty term depends explicitly on the number of parameters, WAIC and LOO incorporate a more intricate calculation. They take into account the effective number of parameters (variance of the log-likelihood, given by equation (2.11)), which depends on the structure and complexity of the model and the influence of the prior distributions. This leads to a more accurate reflection of both the model fit and its generalization capabilities, ensuring that the selected model is not only well-fitted to the current data but also robust for predicting new, unseen data. Notably, the fully Bayesian information criterion WAIC closely approximate LOO cross-validation.

### 3.4. Probabilistic programming languages benchmark

This section focuses on fitting the model using NUTS within several popular Python-based PPLs. We compare their efficiency in terms of computational cost and quality of the inference obtained (convergence of chains and effective sample size). To ensure a fair comparison across PPLs, we ran NUTS with identical settings in each: default NUTS parameters (max tree depth of 10, acceptance rate of 0.8), four parallel chains, initialization in the tails of the prior, and 200 warm‐up and 200 sampling iterations.

**Figure 8.**
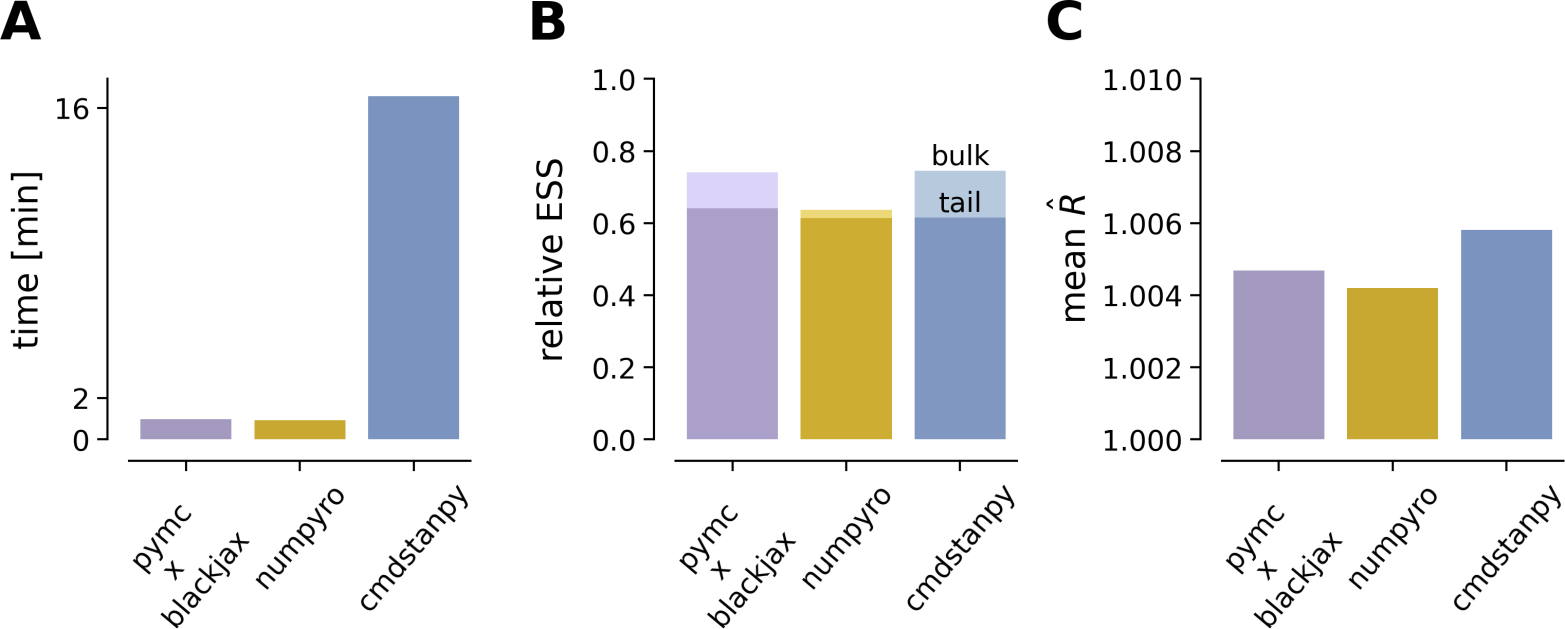
Benchmarking of NUTS implementation in solving an ODE model given by equation (2.1) across different popular PPLs. We report the following metrics for our comparison: (A) Computational time in minutes (four parallel chains, 200 warm‐up steps and 200 samples). (B) Relative effective sample size ESS, i.e. number of effective samples divided by total draws. (C) Mean value of $\hat{R}$ across all model parameters (measure of convergence between and within chains in MCMC, closer to 1 indicates better convergence).

Our implementation reveals that NumPyro and PyMC (using the BlackJAX backend) were approximately 16 times faster than Stan (figure 8A and table 3) achieving virtually identical convergence properties, $\hat{R}\approx 1.00$ and effective sample sizes (see figure 8B,C and table 3). The only notable difference was NumPyro achieving a lower effective sample size in the bulk of the posterior (ESS bulk), that is in the high probability region, compared with the two other samplers (figure 8B and table 3).

**Table 3.** Benchmarking of NUTS implementation in solving an ODE model given by equation (2.1) in several PPLs. NUTS was run with default parameters in each software (maximum tree depth 10 and target acceptance rate 0.8) with 200 warm‐up and 200 collected samples.

package	time (s)	mean ESS		rel. ESS		mean ESS bulk (s)	mean $\hat{R}$	divergences
		bulk	tail	bulk	tail			
NumPyro	55.532	508.89	491.538	0.636	0.614	9.164	1.004	0
PyMC × BlackJAX	59.076	592.603	512.673	0.741	0.641	10.031	1.005	0
CmdStanPy	993.862	596.246	492.626	0.745	0.616	0.600	1.006	0

## 4. Discussion

### 4.1. Related work and implementation in probabilistic programming languages

#### 4.1.1. Related work

In evaluating the performance of various MCMC methods for Bayesian model inversion in DCM, previous research highlighted significant challenges [16,17]. The gradient-free methods investigated in Sengupta *et al*. [16] included random walk Metropolis, slice-sampling, adaptive MCMC and population-based MCMC with tempering. Among these, slice sampling emerged as the most efficient gradient-free algorithm in terms of effective samples, yet it still provided less than 10 effective samples out of 1400 draws (after excluding 600 burn-in samples from an initial 2000). Gradient-based methods, including Hamiltonian Monte Carlo (HMC) and Langevin Monte Carlo (LMC) with two variants—Langevin diffusion on a Euclidean manifold (LMC-E) and a Riemannian manifold (LMC-R)—also faced substantial difficulties [17]. Specifically, LMC-E yielded an effective sample size of only 11 out of 14 000 samples (after excluding 6000 burn-in samples from an initial 20 000), while HMC produced fewer than 100 effective samples despite an extensive run time of 45 h. The computing performance of HMC in this case was voluntarily not optimized to assert for worst-case behaviour.

#### 4.1.2. Implementation in probabilistic programming languages

By leveraging advanced computational techniques and optimized automatic differentiation libraries, HMC can achieve efficient and accurate inference, overcoming the obstacles faced by earlier studies. This provides a more encouraging perspective on the use of Monte Carlo methods in DCM. In this work, we achieved MCMC convergence of four parallel chains using only 200 burn-in samples, and obtained accurate inference in less than 1 min (see table 3). The implementations in NumPyro, PyMC and Stan enabled us to perform very efficient inference with NUTS, achieving an effective sample size that was more than half of the actual number of samples. We note that the slower performance of Stan compared with the other PPLs lies in the non-vectorized Euler integration loop, which accounts for most of the computational time. A more efficient implementation using vectorization could potentially improve the inference in Stan compared with others. While we observed that NumPyro or PyMC (using BlackJAX or Numpyro backends, as shown in electronic supplementary material, figure S7 and table S2) had a computational advantage over Stan with this specific model, it is important to note that this advantage should not be generalized. Our objective was not to conduct a comprehensive benchmark of PPLs across different inference tasks. Previously, the computational efficiency of Stan and NumPyro has been compared across several datasets [95], with results showing that while NumPyro tends to outperform Stan across numerous datasets (approximately 2.3× speed-up), there are instances where Stan performs better. Thus, the performance difference can vary depending on the specific dataset and task.

The final point to mention here is that PyMC provides implementations of a large number of gradient-free and gradient-based MCMC algorithms, as well as sequential Monte Carlo (SMC; [96,97]) algorithms. Consistent with previous studies [16], our results also indicated that NUTS, as gradient-based MCMC algorithm, significantly outperformed gradient-free algorithms such as the Metropolis family (see electronic supplementary material, figure S7). SMC outperformed the aforementioned gradient-free samplers and was significantly faster than NUTS. However, its performance heavily depends on the acceptance threshold value.

#### 4.1.3. Ease of implementation across probabilistic programming languages

The ease of implementation also plays a crucial role in selecting the appropriate PPL for a given task. NumPyro and PyMC share similar high-level concepts and syntax, making them relatively easy to implement for users familiar with probabilistic modelling in Python. Both frameworks rely on a similar approach, where the user defines a probabilistic model (using a model block) and subsequently calls functions for fitting and sampling from the model. One key advantage of NumPyro and PyMC over Stan is that the model definition and sampling process are tightly integrated into the Python ecosystem. Users can define models in pure Python code and can also easily use existing Python libraries to define custom functions or perform additional computations on their models. This flexibility allows for more intuitive interactions and faster prototyping. An additional feature of NumPyro is its integration with JAX, which allows for automatic differentiation and scalable computations on GPUs. This can be advantageous in cases where computational efficiency is crucial. However, this also requires familiarity with JAX, and the model implementation can become more complex if advanced optimizations or custom JAX-based logic are required. CmdStanPy is a Python interface to the Stan modelling language, providing a more direct, lower-level interface to Stan’s powerful sampling and inference capabilities. By contrast to NumPyro and PyMC, CmdStanPy requires the user to write the Stan model in a separate Stan language script. However, CmdStanPy and other Stan interfaces benefit from the large and robust Stan community, which provides extensive resources through the well-established workshops, discourse forum and various communication channels. PyMC and NumPyro both emphasize user-friendly tutorials and have growing communities. Additionally, PyMC offers numerous algorithm implementations and supports multiple backends, including BlackJAX and NumPyro. Nevertheless, once samples are obtained, the outputs of a large variety of PPLs, including Stan, can be easily handled using ArviZ [89], a popular Python library for Bayesian inference that provides visualization tools and diagnostic utilities.

### 4.2. Degeneracy

Degeneracy, the ability of structurally different elements to perform the same function or yield the same output, is a ubiquitous property in biological systems and evident at genetic, cellular, system and population levels [34]. Degeneracy is a fundamental aspect of neural architecture and is crucial for the brain’s resilience and adaptability [36]. By allowing multiple neural circuits or brain regions to compensate for each other, degeneracy ensures that the brain can maintain functionality despite damage or perturbations [37,98]. This (many-to-one mapping) capability is essential for processes such as learning, memory and recovery from injury, where different neural pathways can adapt and take over functions when primary pathways are compromised. However, while degeneracy provides a robust mechanism for maintaining brain function, it poses challenges for inference, such as deciphering a bijective (one-to-one mapping) relationship. The overlapping functionalities of different neural elements can lead to difficulties in pinpointing specific causal pathways and accurately estimating model parameters. This complexity underscores the need for advanced computational methods and robust probabilistic approaches to disentangle the contributions of various neural components and better understand the intricate dynamics of brain function.

The overlapping functionalities inherent in degeneracy add another layer of complexity to the issue of non-identifiability in modelling neural dynamics. Structural non-identifiability [99–101] is a common issue in partially observed dynamical systems described by ODEs. For a model to be structurally identifiable, it must allow for a unique set of parameters for any given output, ensuring that the solution to the inverse problem is not only unique but also depends continuously on the data. This well-posedness is crucial from a statistical viewpoint, as it enables robust parameter estimation, even with minor data perturbations [102]. Given the often imprecise nature of data, we should expect these estimates to remain relatively stable with small changes. This stability is crucial for reliable inference and model validation. However, ill-posedness arises when the reconstruction of causes from observed effects becomes unstable, meaning that vastly different causes could produce similar effects. This poses significant challenges for accurately identifying causal relationships in complex systems. Structural identifiability issues stem from the model’s inherent design, which can render some parameters indeterminable (see electronic supplementary material, figure S8, where, in the present case, the connectivity parameter $g_{1}$ shows clear structural non-identifiability). By contrast, practical identifiability challenges occur when the available measurements lack sufficient information to determine the parameters with the required precision. In the present model, profile likelihood methods [99,100,103] suggest the practical non-identifiability of input parameter u beyond a certain threshold (see electronic supplementary material, figure S8). In inference, degeneracy manifests as a lack of information, complicating the extraction of precise and reliable parameter estimates (as we observed in figure 2, where inference converges to several modes of parameter space, failing to recover the underlying dynamics of the data). The stacking of predictive distributions from multiple MCMC chains that we employed as a remedy (see figure 5) leverages their combined information, thereby enhancing the overall information content and addressing the challenges posed by degeneracy. Initializing chains within prior ranges (see figure 4) is another solution that incorporates additional information, effectively mitigating the degeneracy encountered during inference on ODE models.

### 4.3. Initialization at the tails

An intuition behind initializing in the tails is that it will generalize better when scaling to higher dimensions than initializing in regions of high probability. Counterintuitive to our usual representations of one- or two-dimensional, often Gaussian distributions, which place the largest amount of probability mass around the mode, this does not hold in high dimensions. In fact, most of the mass of the standard Gaussian distribution in a high dimension lies in its tails [104]. Hence, the typical set explored by methods like HMC will not include the volume around the mode [105]. Such behaviour arises from the unusual geometry of high-dimensional spaces: the volume of a high-dimensional ball is concentrated in its crust [104]. For example, when uniformly sampling points within a high-dimensional unit hypercube, most samples will fall in the corners of the hypercube, outside the inscribed hypersphere [105]. It remains to define high dimension: a 10-parameter set as explored in this work may not fully reflect this phenomenon; however, Carpenter [105] shows that at 10 dimensions, the volume of the hypersphere inscribed in the unit hypercube has almost vanished.

### 4.4. Variational inference

The mean-field approximation, while computationally efficient, is known to underestimate marginal variances [72], as we also observed in our results for a DCM of ERPs (see figure 6). The simplification inherent in the mean-field approach can lead to overly confident parameter estimates. By contrast, full-rank approximation mitigates this issue by capturing correlations between variables, thus providing a more accurate representation of the posterior distribution. Similarly, the Laplace approximation, when applied without the mean-field assumption, has shown impressive results, producing posterior inferences that are competitive with those obtained via HMC. Based on analytical derivations, this method combines advantageous computational efficiency with robust inferential accuracy, making it a viable alternative to more computationally intensive techniques like HMC. Note that, in our comparisons, we ran the Laplace VI with $10^{5}$ iterations to ensure consistency in the benchmark with other algorithms. However, the Laplace method converges quickly, and its computational time can be significantly reduced through early stopping, without compromising the quality of the results.

The potential downsides of the Laplace approximation are due to its purely local nature, depending solely on the curvature of the posterior around the optimum [62]. Still, the most common advice when working with difficult posterior geometry is to reparametrize the model to obtain a log convex posterior [57,106]. The Laplacian approximation may still therefore be widely applicable. A second computational constraint of the Laplace approximation is the need to invert the Hessian matrix of second-order derivatives, which has complexity $O(n^{3})$ with the number of parameters. As with posterior geometry, reparametrization can allow structuring the Hessian into mean and full‐rank components, to sidestep costly large matrix inversions.

One of the core advantages of ADVI is that it removes the need for manual tuning by the user. In ADVI, the main idea is to represent the gradient as an expectation, and to use Monte Carlo techniques to estimate this expectation. Although ADVI leverages Gaussian mean-field and full-rank distributions, it operates with transformed variables, which induce non-Gaussian variational distributions in the original variable space. Ultimately, ADVI aims at enabling a generic inference algorithm, functioning as a black-box, without requiring analytical computation of the ELBO or assumptions about the variational family.

Beyond these methods, traditional variational inference typically minimizes the variational lower bound using the KL divergence [107,108]. However, recent advancements have explored the use of alternative divergence measures, such as the Rényi divergence [109–111]. This approach introduces a hyperparameter that, when set to 1, reduces to the KL divergence. By adjusting this parameter, the approximation can shift between mode-seeking behaviour, which focuses on identifying the highest probability regions (but includes variability and is not reduced to point estimates), and mass-covering behaviour, which aims to cover the entire distribution more comprehensively. We note that the ELBO objective leveraging Rényi divergence is implemented in NumPyro. Investigations into these types of alternatives in variational inference methods or inference networks [68,112,113] were beyond the scope of this study and remain to be explored in future research.

### 4.5. Model comparison

We conducted a comprehensive model comparison in both VI and MCMC frameworks. Variational approximation typically relies on maximizing the ELBO, which equivalently minimizes the free-energy, to assess model performance and compare different models. The ELBO (or negative free-energy) provides an estimate of model evidence that balances model fit and complexity. In the MCMC framework, model comparison works in an analogous way and is based on quantities computed post hoc and without additional cost [84,85], such as classical AIC [80] and BIC [81], or the more recently developed WAIC [86,87] and LOO [85], which are tailored to the Bayesian context [84]. Our results showed that model comparisons based on AIC, BIC, WAIC and LOO in the MCMC framework aligned closely with free-energy from variational inference (figure 7). For researchers accustomed to variational inference, this finding provides reassurance that model comparison can be seamlessly transitioned to the MCMC framework without losing consistency in evaluation.

By definition, classical information criteria (AIC/BIC) are agnostic to prior information as they rely on maximum likelihood estimation, and the penalty term is determined solely by the number of parameters and observed data. By contrast, fully Bayesian information criteria (WAIC) and LOO cross-validation enable the incorporation of prior information into the model’s fit to data, thereby improving out-of-sample prediction accuracy through the integration of (clinical or biological) knowledge. This approach has been used in medical applications such as identifying epileptogenic zones in the brain [32] or predicting drug plasma concentration in alcohol use disorder [94]. Notably, the Bayesian LOO expected log point density approximates classical LOO cross-validation, which avoids the need to repeat the fitting process, making it a powerful and efficient estimate especially in contexts where fitting models can be computationally expensive. Moreover, WAIC closely approximates Bayesian LOO, indicating a direct link between information theory and cross-validation [84,85].

### 4.6. Limitations and future research

This work focused on synthetic data to validate the algorithms rather than directly validating the model against empirical data. We limited our investigation to a single neural mass model, which may not fully capture the complexities of more elaborate DCM models involving intricate neural networks at larger scales. Extending our analysis to whole-brain‐level DCM presents a significantly more complex inversion problem that may necessitate the incorporation of sparse priors or other regularization techniques to obtain meaningful results. Our current work focused on ODE state-space models, but estimating dynamic and observation noise for fMRI data poses additional challenges for convergence. Investigating MCMC and variational inference methods for the DCM family operating at higher dimensional model configurations subject to noise remains an area of future research.

As network size increases, the computational cost of model inversion grows; however, convergence of gradient-based methods such as HMC and Laplace may be hindered by geometric challenges and numerical issues in high-dimensional parameter spaces. To avoid these challenges, likelihood-free methods and recursive filtering approaches have been applied at different network scales. For instance, Tolley *et al*. [114] have provided guidelines and considerations for using simulation-based inference (SBI; [115,116]) to investigate the cellular and circuit origins of commonly measured MEG/EEG signals, including low-frequency oscillations (e.g. beta events and ERPs), along with differences across experimental conditions. In higher-dimensional settings, Zhao *et al*. [117] have developed a semi-analytical Kalman filter approach to fit uncoupled neural mass models to each electromagnetic source time series, enabling scalable inference across multiple sources. Lu *et al*. [118] used the ensemble Kalman filter to fit spiking neuronal networks, at the large-neuron scale of the human brain, to empirical BOLD data. The scalability of both MCMC-based approaches and SBI to whole-brain models has already been demonstrated in previous studies. Hashemi *et al*. [93] and Jha *et al*. [106] have shown that, with appropriate reparametrization techniques, NUTS scales to whole-brain models of epilepsy spread. Hashemi *et al*. [101,119], Sorrentino *et al*. [52] and Ziaeemehr *et al*. [120] have developed scalable SBI tools to estimate individualized parameters from whole-brain stereo-EEG, MEG/EEG and BOLD recordings. The results of these studies indicate that the choice of model inversion method—and its scalability—depends on several factors, including the complexity, sensitivity and stiffness of the underlying neural mass equations, as well as the level of dynamical noise, coupling strength and network effects. A comprehensive benchmark evaluating the scalability of popular inversion techniques for brain network models remains to be investigated in future studies.

## Data Availability

All code is available on GitHub (https://github.com/ins-amu/DCM_PPLs), Zenodo [121] and in Ebrains collaboratory drive task 3.3. Supplementary material is available online [122].
